# Simple Algorithm for Blood Morphology Analysis (SAMBA): An Artificial Intelligence-Assisted Approach to Identify Peripheral Blood Phenotypes in a Cohort of Patients with Pancreatic Ductal Adenocarcinoma (PDAC)

**DOI:** 10.3390/cancers18213477

**Published:** 2026-10-28

**Authors:** Aleksander Wardęszkiewicz, Konrad Kosztowny, Oliwia Grząsiak-Kraj, Piotr Hogendorf, Adam Durczyński, Anna Kumor-Kisielewska, Michał Panek, Janusz Strzelczyk, Alicja Majos

**Affiliations:** 1 Department of General and Transplant Surgery, Norbert Barlicki University Hospital, Medical University of Łódź, 90-153 Łódź, Poland; aleksander.wardeszkiewicz@umed.lodz.pl (A.W.); konrad.kosztowny@umed.lodz.pl (K.K.); piotr.hogendorf@umed.lodz.pl (P.H.); adam.durczynski@umed.lodz.pl (A.D.); janusz.strzelczyk@umed.lodz.pl (J.S.); alicja.majos@umed.lodz.pl (A.M.); 2 Department of Pulmonology, Norbert Barlicki University Hospital, Medical University of Łódź, 90-153 Łódź, Poland; anna.kumor@umed.lodz.pl; 3 Department of Internal Medicine, Asthma and Allergy, Norbert Barlicki University Hospital, Medical University of Łódź, 90-153 Łódź, Poland

**Keywords:** cancer, inflammation, type 2 inflammation, eosinophils

## Abstract

Introduction: Eosinophils have become important therapeutic targets in several allergic diseases. This study aimed to apply a similar approach—using peripheral blood findings to identify features of type 2 (T2) inflammation—to oncology. We used Simple Algorithm for Blood Morphology Analysis (SAMBA) to describe blood count profiles in a cohort of patients with PDAC independently of relative ratios and propose a potential classification. Materials and Methods: We included 153 treatment-naïve patients with PDAC confirmed by postoperative histopathology. Of these, n = 68 (44.4%) underwent curative surgery, whereas n = 85 (55.6%) received palliative treatment or underwent surgical biopsy only. SAMBA was applied using laboratory reference ranges and overall survival (OS; months) as the analytical context. Results: The subgroups defined according to laboratory reference ranges did not differ significantly in disease stage, grade, or resection status. Based on correlation analyses, Kaplan–Meier curves, and a classification and regression tree (CART) analysis of OS, we propose four blood count phenotypes: exhausted, lymphopenic, T2-low, and T2-high (median OS: 1.5 months, n = 22; 4.5 months, n = 12; 8.0 months, n = 25; and 10.0 months, n = 94, respectively). Conclusions: We propose the following preliminary interpretations of these phenotypes: exhausted, potentially associated with concomitant infection or marked inflammation; lymphopenic, characterized by a low lymphocyte count and potentially reduced antitumor immune activity; eosinopenic (T2-low), characterized by a relatively balanced inflammatory profile but limited T2-associated features; and eosinophilic (T2-high), characterized by no major abnormalities in the other blood count parameters and a more favorable prognosis. SAMBA is a transparent, readily interpretable algorithm that may help standardize and accelerate preliminary research in immuno-oncology.

## Simple Summary

In recent years, the recognition of eosinophils as the key cells in different allergic diseases has made them the target of therapeutic strategies. The aim of this study was to transfer this way of thinking—focused on researching features of T2 inflammation—to the field of oncology to describe blood count profiles of a PDAC patient cohort in a non-relative manner (Simple Algorithm for blood Morphology Analysis, SAMBA) and propose a potential classification. Based on correlation analysis, Kaplan–Meier curves and a decision tree (CART) analysis of overall survival (OS), we propose a classification of blood count phenotypes: exhausted, lymphopenic, T2-low and T2-high (median OS: 1.5, n = 22; 4.5, n = 12; 8.0, n = 25; 10.0, n = 94). A transparent and easy-to-interpret algorithm may unify and accelerate preliminary research in the field of immuno-oncology.

## 1. Introduction

Pancreatic ductal adenocarcinoma (PDAC) poses a multifaceted clinical challenge. Although surgical resection may be possible in selected patients, treatment remains difficult. PDAC is often described as an immunologically “cold” tumor, yet it can also be associated with paraneoplastic hypereosinophilia. It remains challenging to treat, including with chemotherapy, and is resistant to single- and dual-checkpoint inhibition [1]. Current and emerging approaches to PDAC treatment include immunologically based strategies such as vaccines, adoptive lymphocyte transfer, and cachexia-directed therapies [2,3]. Optimizing these approaches requires careful patient selection. Various inflammatory markers and indices have been developed and evaluated, but they have not become established tools in routine clinical oncology. These include the neutrophil-to-lymphocyte ratio (NLR; neutrophil count divided by lymphocyte count), lymphocyte-to-monocyte ratio (LMR; lymphocyte count divided by monocyte count), monocyte-to-lymphocyte ratio (MLR; the inverse of LMR), platelet-to-lymphocyte ratio (PLR; platelet count divided by lymphocyte count), systemic immune-inflammation index (SII; platelet count multiplied by NLR), and systemic inflammation response index (SIRI; neutrophil count multiplied by the monocyte-to-lymphocyte ratio). These indices are inexpensive, readily available, and repeatable. However, their apparent discriminatory value for progression-free survival or overall survival may reflect their ability to identify patients with markedly disturbed homeostasis (e.g., concurrent lymphopenia and neutrophilia), which may already be apparent from the individual counts. Consequently, these indices may not capture more subtle differences between patients with favorable and unfavorable prognoses. If blood counts reflect systemic inflammation to some extent and may reveal cancer-associated inflammatory patterns, simply constructing an index may not be sufficient to establish clinical utility [4].

In recent years, the identification of eosinophils as key effector cells in several allergic diseases has led to their use as therapeutic targets. Peripheral blood eosinophil counts have become an important biomarker in asthma classification and in selecting patients for biologic therapy. In a previous study, we applied a similar approach to an oncology cohort by investigating blood-based evidence of T2 inflammation or its absence. We found a positive association between the absolute eosinophil count and overall survival in patients with PDAC [5]. This approach may also inform the development of new treatment strategies, although translating an observed association with an uncertain mechanistic basis into a therapeutic discovery requires substantial further research. Several meta-analyses published in 2026 reported consistent associations between inflammatory indices and cancer outcomes:

- PDAC: An elevated SII was associated with poorer overall survival [6].
- Triple-negative breast cancer: The NLR was associated with pathological complete response (OR = 5.61, 95% CI: 2.05–15.34; *p* = 0.000) [7].
- Head and neck cancer: A high SIRI was associated with poorer overall survival [8].
- Melanoma: The NLR was associated with poorer survival (HR = 2.34, 95% CI: 1.86–2.93) [9].
- Oral squamous cell carcinoma: A high pretreatment NLR was associated with poorer OS (HR = 1.61) and disease-free survival (DFS; HR = 1.64) [10].
- Head and neck cancer: A high NLR consistently predicted poorer OS [11].
- Esophageal cancer: The NLR was significantly associated with OS (HR = 2.01, 95% CI: 1.64–2.45) [12].
- Lung cancer treated with PD-1/PD-L1 inhibitors: An elevated NLR was associated with shorter OS (HR = 1.92, 95% CI: 1.70–2.17) and progression-free survival (PFS; HR = 1.66, 95% CI: 1.48–1.87) [13].

A common limitation of the studies listed above is that they focus on ratios rather than the raw values of the component parameters. This makes it difficult to characterize the nature and potential mechanisms of the observed associations and may hinder further investigation. The present study extends this approach to the remaining major white blood cell populations and describes blood morphology profiles in patients with PDAC independently of relative ratios, using our Simple Algorithm for Blood Morphology Analysis (SAMBA). We also propose a classification of peripheral blood morphology patterns for further investigation.

## 2. Materials and Methods

The inclusion criteria were age ≥ 18 years and histopathological confirmation of PDAC in postoperative material. The exclusion criteria were percutaneous biopsy without surgical material, unavailable analytical data, a second active cancer, corticosteroid use within 8 weeks before the procedure, and preoperative chemotherapy. In total, 153 treatment-naïve patients with PDAC confirmed in postoperative material were included, of which 68 (44.4%) underwent curative surgery, whereas 85 (55.6%) received palliative treatment or underwent surgical biopsy only. We collected data on age, sex, tumor location, disease stage (TNM classification according to the 8th edition of the American Joint Committee on Cancer staging system), tumor grade, blood morphology parameters (eosinophil count [EOS], monocyte count [MONO], neutrophil count [NEU], basophil count [BASO], and lymphocyte count [LYMPH]), and overall survival (OS). OS was defined as the time from surgery to death or the end of follow-up (censored observation). Patients who underwent surgery between 2013 and 2019 were included, and follow-up continued until 1 March 2020. C-reactive protein (CRP) levels measured within 24 h before surgery were also collected and analyzed. CRP data were incomplete because this test was not routinely required before surgery in patients considered free of acute infection, whereas a blood count was required to assess hemoglobin levels. Table 1 presents the general characteristics of the study cohort.

Blood counts were obtained from samples collected within 24 h before surgery. Samples were processed using an automated hematology analyzer (Sysmex XN-2000; Sysmex, Warsaw, Poland), which provided a complete blood count with differential, including the absolute eosinophil count. Results were expressed as absolute cell counts (G/L). The laboratory reference ranges were as follows: NEU, 1.8–7.5 G/L; LYMPH, 1.0–4.5 G/L; MONO, 0.1–1.1 G/L; EOS, 0.05–0.50 G/L; BASO, ≤0.15 G/L; CRP, <4 mg/L; CA 19-9, <37 U/mL; CA 125, <35 U/mL; CEA, <10 U/mL; and CA 15-3, <30 U/mL. Results within the reference ranges are referred to as “optimal” in this study.

**Figure 1:**
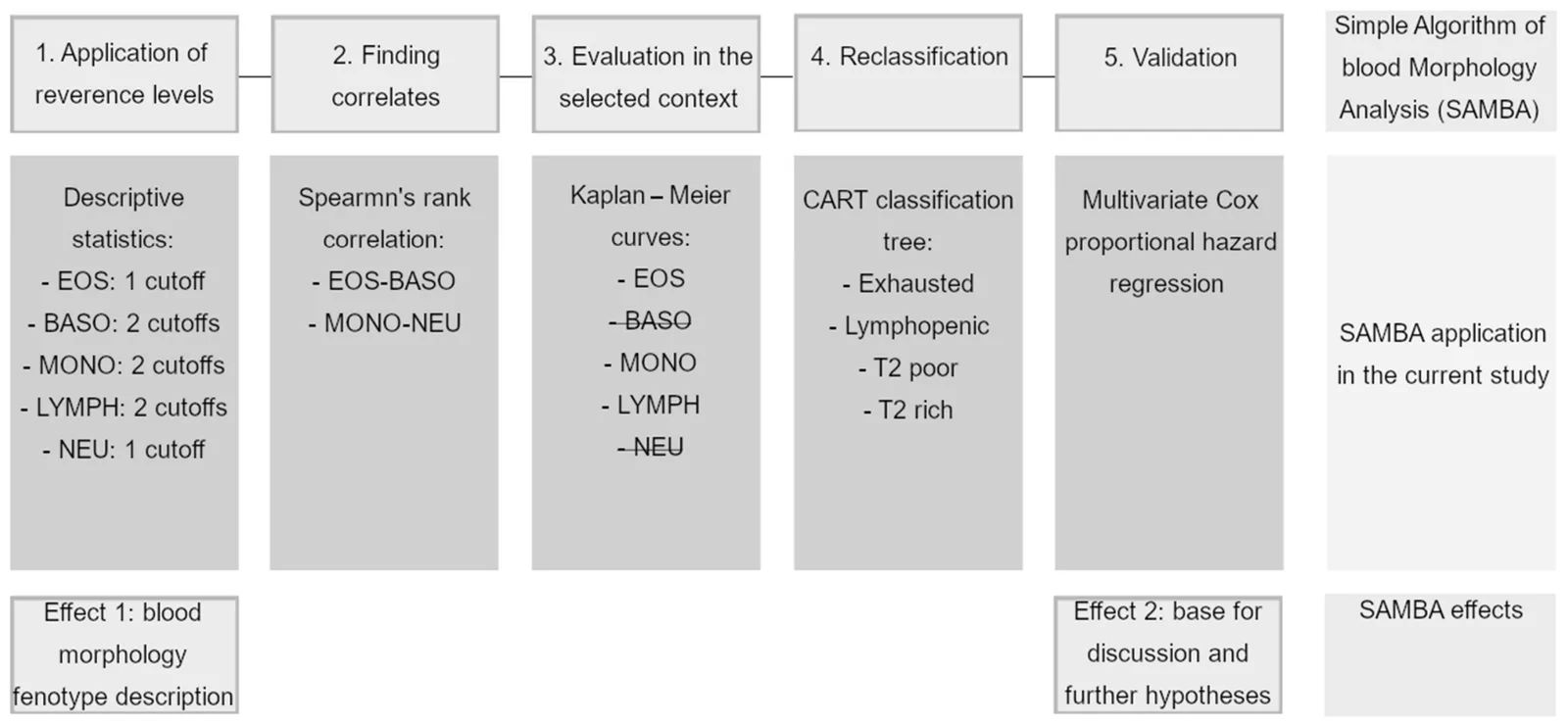
Simple Algorithm for Blood Morphology Analysis (SAMBA), illustrating its application in the present study (**middle**) and its proposed outputs (**bottom**). Cross-outs symbolize removing variable from further analysis.

Statistical analyses were performed using Statistica version 13.1 and Numiqo on-line software (v2026 available at: https://numiqo.com accessed on 5 September 2026). Categorical variables were compared using the chi-square test, and continuous variables were analyzed using the Mann–Whitney U test or the Kruskal–Wallis test with Bonferroni correction. Median survival was estimated using the Kaplan–Meier method. There were 141 deaths; 12 observations were censored. Differences between survival curves were evaluated using the log-rank test or its extension for multiple-group comparisons. CRP was the only variable with missing data and was therefore excluded from multiple comparisons; it was analyzed as a continuous variable in univariate analyses, with records containing missing values excluded. Cox proportional hazards regression was used for survival analyses. The proportional hazards assumption was assessed by examining Schoenfeld residuals over time. All categorical variables were included in univariate and multivariate analyses. Statistical significance was defined as *p* < 0.05. Because developing an evidence-based classification presents methodological challenges, we designed and applied SAMBA (Figure 1). SAMBA comprises standardized steps involving input variables, decision rules, thresholds, and final phenotype classification criteria.

1. Application of reference ranges to white blood cell counts. We defined thresholds using the laboratory reference ranges, although literature-based cutoffs may also be used. The dependent variable should reflect the main outcome of interest.
2. Identification of correlated variables to avoid including redundant information in subsequent analyses.
3. Evaluation in the selected clinical context. In this study, overall survival was the primary endpoint; alternatives may include progression-free survival or the annual number of exacerbations. Other variables should be analyzed to provide context and assess predictor independence.
4. Reclassification. At this stage, classification and regression tree (CART) analysis—a statistical method that can support artificial intelligence-assisted classification—is used to summarize previous findings in the context of descriptive statistics and the literature. The approach is intended to examine assumptions consistent with or informed by expert knowledge. In this study, “validation” refers to evaluating the proposed classification concept, particularly the CART output, rather than independently validating the results. Where possible, the classification should be tested using raw data. Here, Cox proportional hazards regression was used to assess the prognostic relevance of the resulting classification. External validation is recommended whenever an appropriate independent dataset is available.

As described above, SAMBA was implemented in Statistica version 13.1 using white blood cell counts as input variables, laboratory reference ranges as thresholds, and OS (months) as the analytical context. We selected CART because it is straightforward to use, is available in the STATISTICA version 13.1 package (unlike some survival-tree methods), and does not require programming skills. The high proportion of observed deaths (92.1% of observations) also supported this choice. CART can be applied in various settings, particularly to predict categorical outcomes. The phenotype assessment was an internal exploratory analysis, not an independent validation. CART parameters were as follows: maximum number of nodes, 6; minimum terminal-node size, 10; candidate predictors, LYMPH, MONO, and EOS; missing data, none; continuous dependent variable, OS time; splitting criterion, Gini coefficient; and misclassification costs, equal. The software program did not provide an assessment of model stability. We evaluated the results using conventional methods (Kaplan–Meier analysis and Cox proportional hazards regression; all reported hazard ratios [HRs] were derived from Cox models) to assess their relevance independently of CART. We also examined the raw data to check the algorithm-derived findings and reduce the risk of errors or misleading outputs.

This study was conducted in accordance with the Declaration of Helsinki and was approved by the Ethics Committee of the Medical University of Łódź, Poland (protocol code: KE/826/23, 10 October 2023).

## 3. Results

We compared clinical characteristics among subgroups defined by laboratory reference ranges (eosinophils, EOS ≤ 0.05 vs. >0.05; basophils, BASO ≤ 0.02 vs. >0.02 and ≤0.06, vs. >0.06; monocytes, MONO ≤ 0.3; vs. >0.3 and ≤0.8, vs. >0.8; lymphocytes, LYMPH ≤ 1.1, vs. >1.1 and ≤2.5, vs. >2.5; neutrophils, NEU ≤ 6.1 vs. >6.1; Table 2, Table 3 and Table 4; post hoc comparisons in Tables S1 and S2 in the Supplementary Materials). We found no statistically significant differences in disease stage, grade, or resection status. We also examined 6- and 12-month survival.

Six-month survival was higher in the MONO 0.31–0.81 G/L subgroup than in the subgroups with MONO < 0.31 G/L or >0.81 G/L (*p* = 0.041; Table 3). Six- and twelve-month survival were both lower in the LYMPH < 1.1 G/L subgroup than in the LYMPH 1.1–2.5 G/L and >2.5 G/L subgroups (*p* = 0.044 and *p* = 0.011, respectively; Table 3). Six- and twelve-month survival were both higher in the NEU ≤ 6.1 G/L subgroup than in the NEU > 6.1 G/L subgroup (*p* = 0.014 and *p* = 0.027, respectively; Table 4).

CRP levels also differed between subgroups. CRP was the lowest in the MONO 0.31–0.81 G/L subgroup compared with the MONO < 0.31 G/L and >0.81 G/L subgroups (median, 3.8 mg/L vs. 12.4 mg/L and 37.7 mg/L, respectively; *p* = 0.041; Table 3). In the LYMPH < 1.1 G/L subgroup, the median CRP level was 3.8 mg/L, compared with 12.5 mg/L in the LYMPH 1.1–2.5 G/L subgroup and 3.0 mg/L in the LYMPH > 2.5 G/L subgroup (*p* = 0.041; Table 3). Median CRP levels were 4.1 mg/L in the NEU ≤ 6.1 G/L subgroup and 10.6 mg/L in the NEU > 6.1 G/L subgroup (*p* = 0.020; Table 4). CA 125 levels were lower in the EOS ≤ 0.05 G/L subgroup than in the EOS > 0.05 G/L subgroup (median, 15.9 U/mL vs. 19.4 U/mL; *p* = 0.044; Table 2).

Characteristics of subgroups defined by literature-based NLR and MLR cutoffs are also shown in Table 4. Six-month survival was lower in the NLR > 5 subgroup than in the NLR ≤ 5 subgroup (*p* = 0.006), whereas the difference in 12-month survival was not statistically significant (*p* = 0.072). Six-month survival was also lower in the MLR > 0.3 subgroup than in the MLR ≤ 0.3 subgroup (*p* = 0.041). Overall, MONO levels of 0.31–0.81 G/L, LYMPH levels > 1.1 G/L, and NEU levels ≤ 6.1 G/L were associated with better survival (SAMBA step 1).

**Figure 2:**
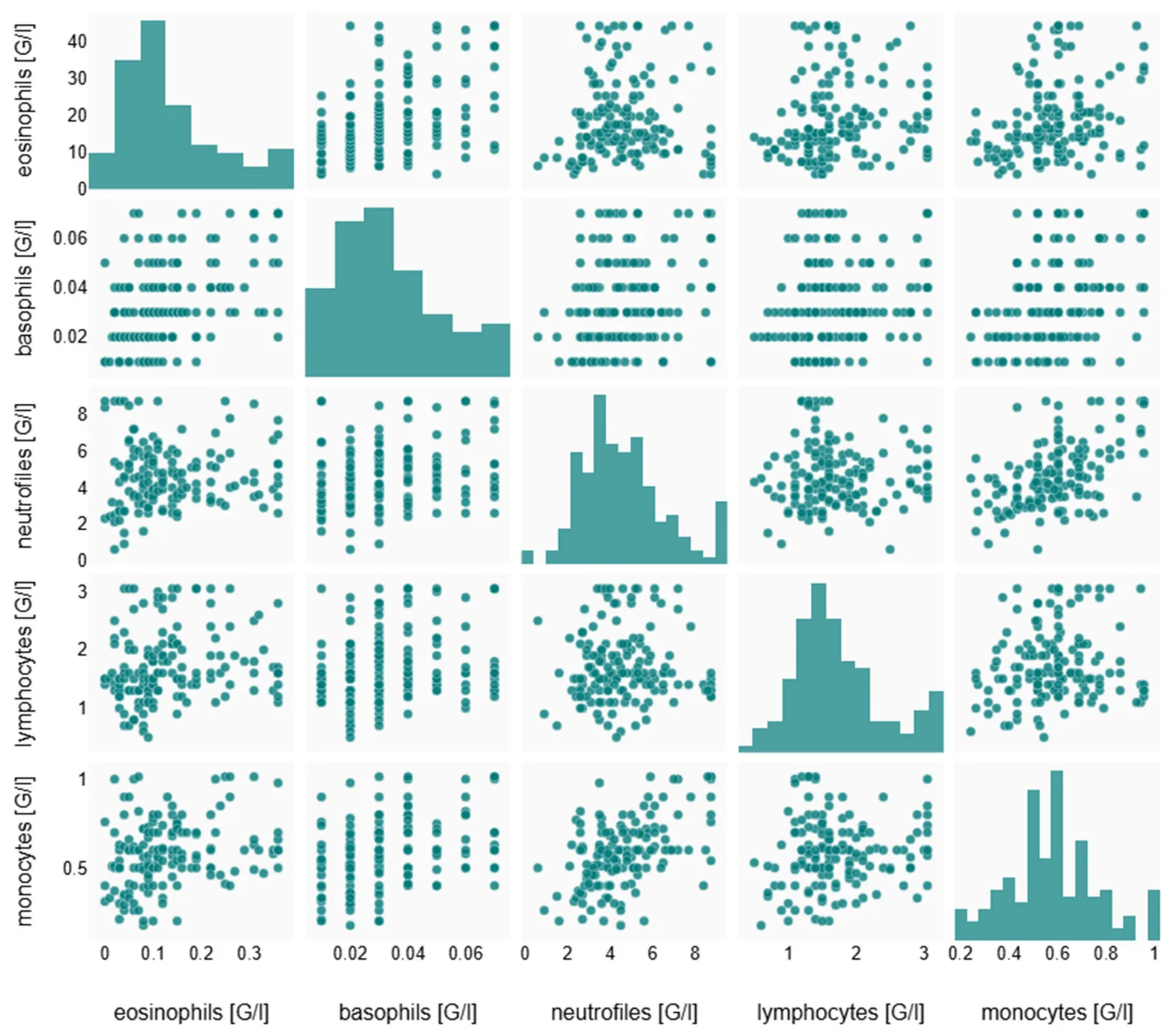
Distribution of blood morphology parameters in relation to each other. Correlation coefficients and *p*-values are shown in Table 5.

We then examined correlations between continuous blood morphology parameters (NEU and MONO: r = 0.46, *p* = 0.000; EOS and BASO: r = 0.48, *p* = 0.000; Table 5 and Figure 2) to avoid including correlated variables in subsequent analyses (SAMBA step 2). BASO was excluded because its association with CRP was similar to that of EOS. NEU and MONO were retained because their relationships with CRP were not consistent, suggesting that they may reflect different underlying processes.

**Figure 3:**
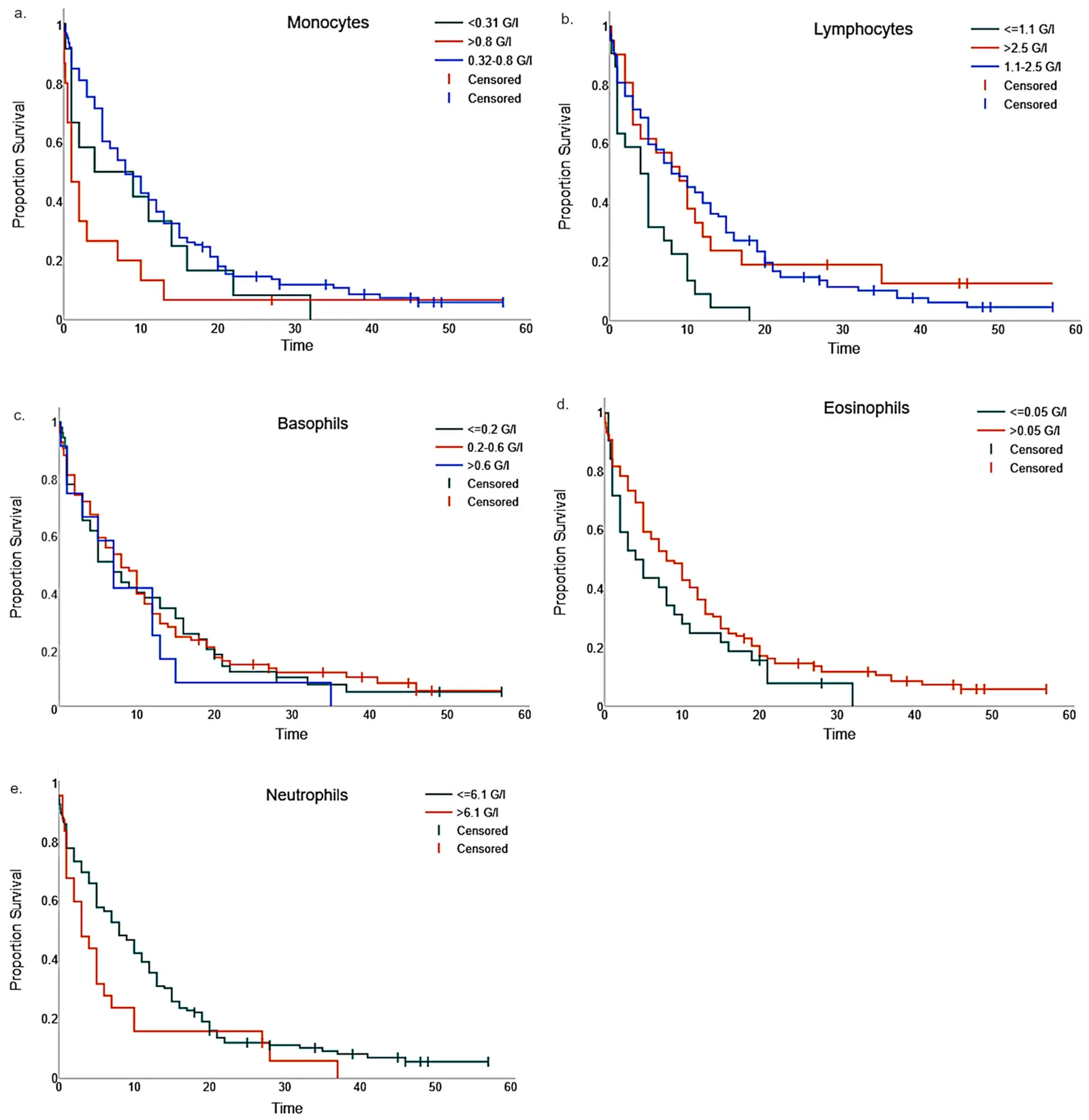
Kaplan–Meier curves for the analyzed variables: (**a**) MONO, *p* = 0.040; (**b**) LYMPH, *p* = 0.025; (**c**) BASO, *p* = 0.885; (**d**) EOS, *p* = 0.146; and (**e**) NEU, *p* = 0.108.

Kaplan–Meier curves were used to evaluate survival in the selected context (OS; SAMBA step 3; Figure 3). Survival curves differed significantly between MONO subgroups (*p* = 0.040) and LYMPH subgroups (*p* = 0.025). The most favorable outcomes were observed in the intermediate MONO range (0.31–0.81 G/L) and the LYMPH range of 1.1–2.5 G/L, whereas outcomes were less favorable at the lower and higher extremes. Visual inspection suggested that BASO did not discriminate between survival curves, while the EOS and NEU curves initially differed but crossed at approximately 20 months of follow-up.

**Figure 4:**
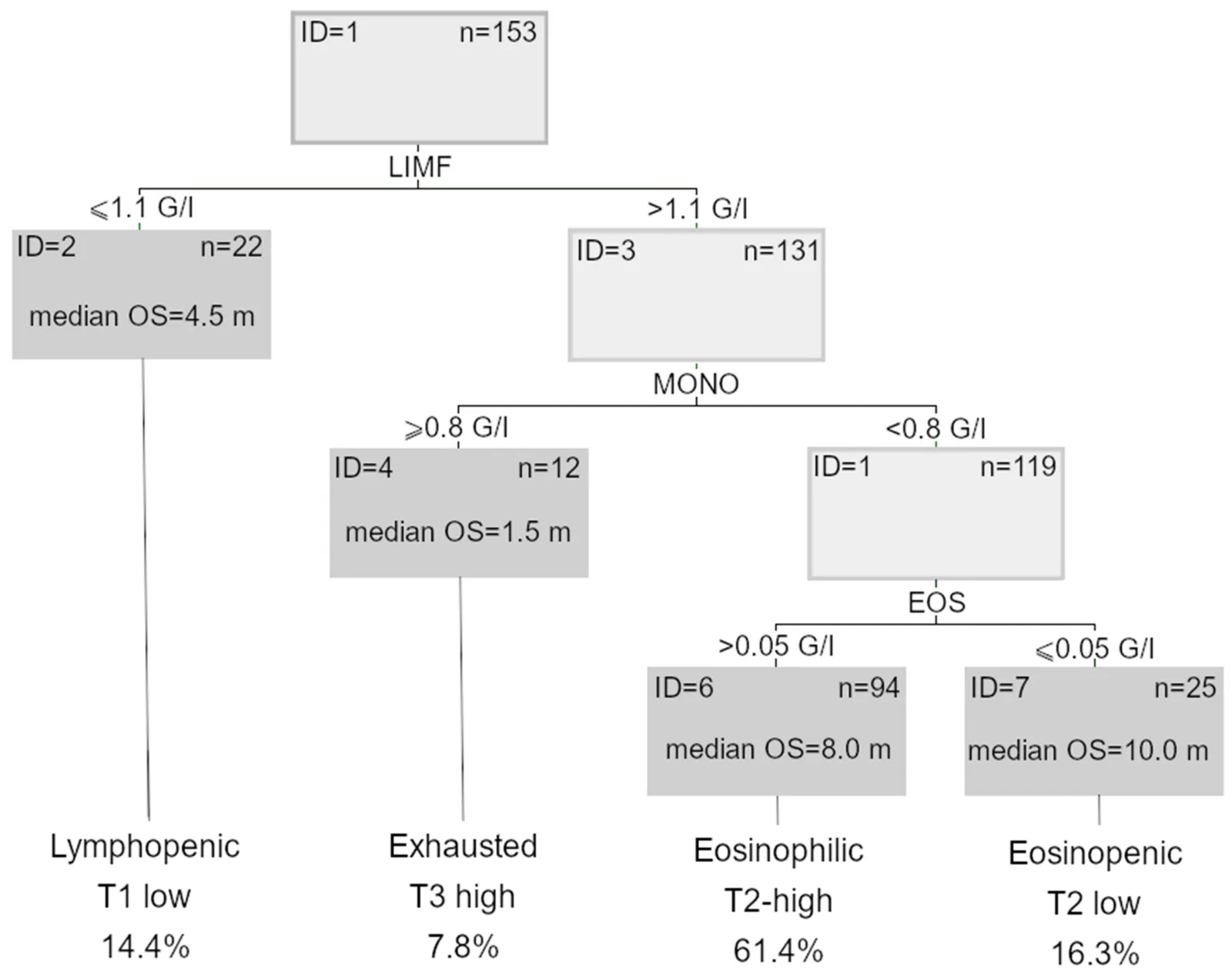
Classification and regression tree (CART). Model parameters: maximum number of nodes, 6; continuous dependent variable, survival time. The labels at the bottom show the proposed nomenclature based on the previous literature, the proposed nomenclature based on the EAACI classification of cell-mediated immune reactions, and the percentage of the study cohort in each group.

**Figure 5:**
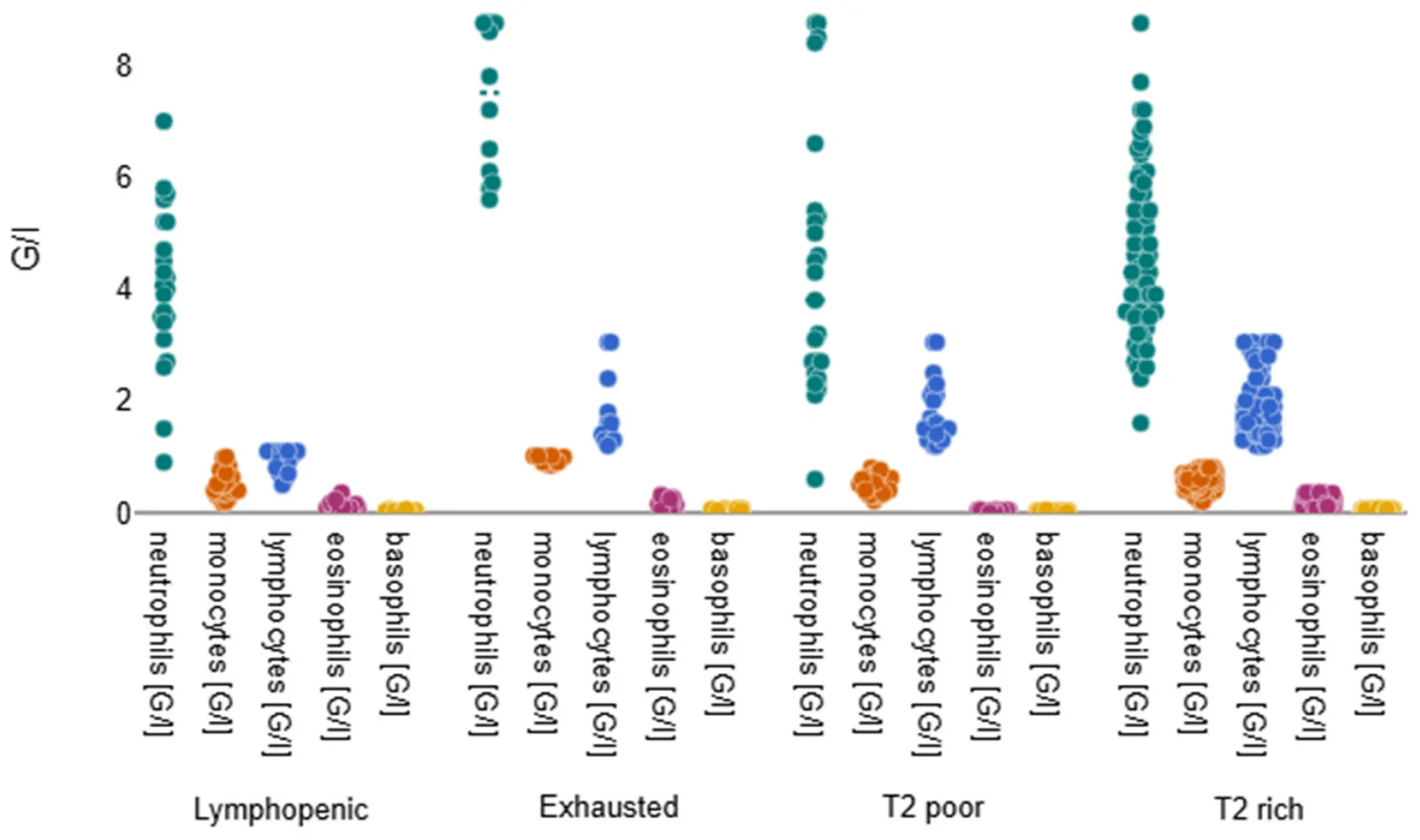
Graphical comparison of blood morphology parameters across the proposed phenotypes (data shown in Table 6). Squares indicate medians, boxes indicate interquartile ranges (IQRs), and whiskers indicate minimum and maximum values. The y-axis is logarithmic; units are G/L.

**Figure 6:**
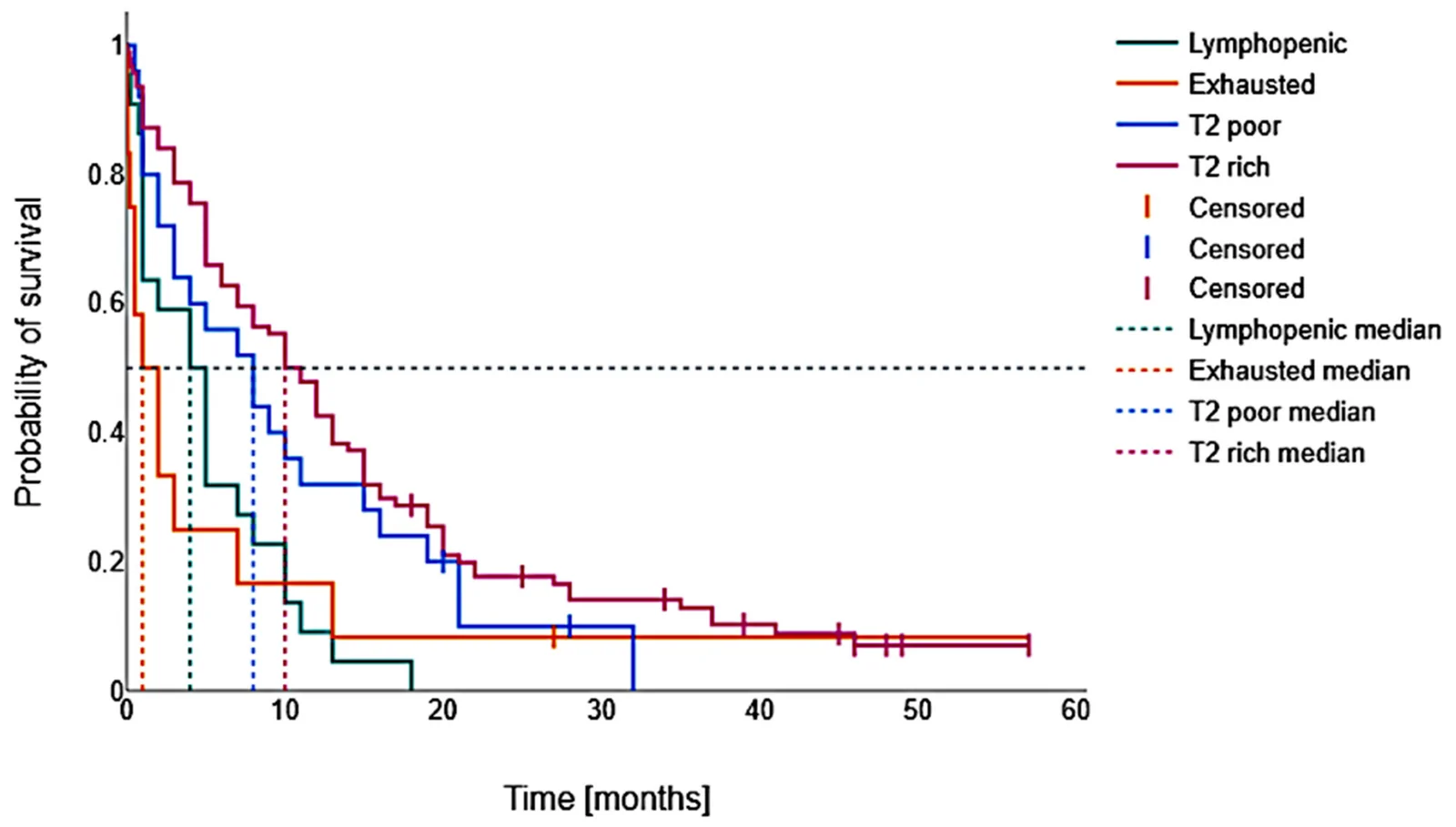
Kaplan–Meier survival curves for the proposed blood morphology phenotypes (*p* = 0.000).

To integrate the preceding findings, we used a CART model for OS (SAMBA step 4). We propose four potential blood morphology phenotypes: exhausted, lymphopenic, T2-low, and T2-high (median OS: 1.5, 4.5, 8.0, and 10.0 months, respectively; n = 22, 12, 25, and 94, respectively; Figure 4). We compared the clinical characteristics of these phenotypes (Table 6; blood cell counts are shown in Figure 5). Although the clinical characteristics did not differ significantly between phenotypes, OS differed significantly. Univariate Cox proportional hazards regression identified stage IV disease, the exhausted and lymphopenic phenotypes, LYMPH ≤ 1.1 G/L, MONO > 0.8 G/L, NLR > 5, and CA 19 – 9 and CA 125 levels as significantly associated with the risk of death (Table 7). In multivariate Cox proportional hazards analyses (SAMBA step 5), the blood morphology phenotype remained an independent prognostic factor for OS in both the full model (Table 8) and the best-fitting model including stage and CA 19 – 9 level (Table 9 and Figure 6; *p* = 0.000).

## 4. Discussion

**Figure 7:**
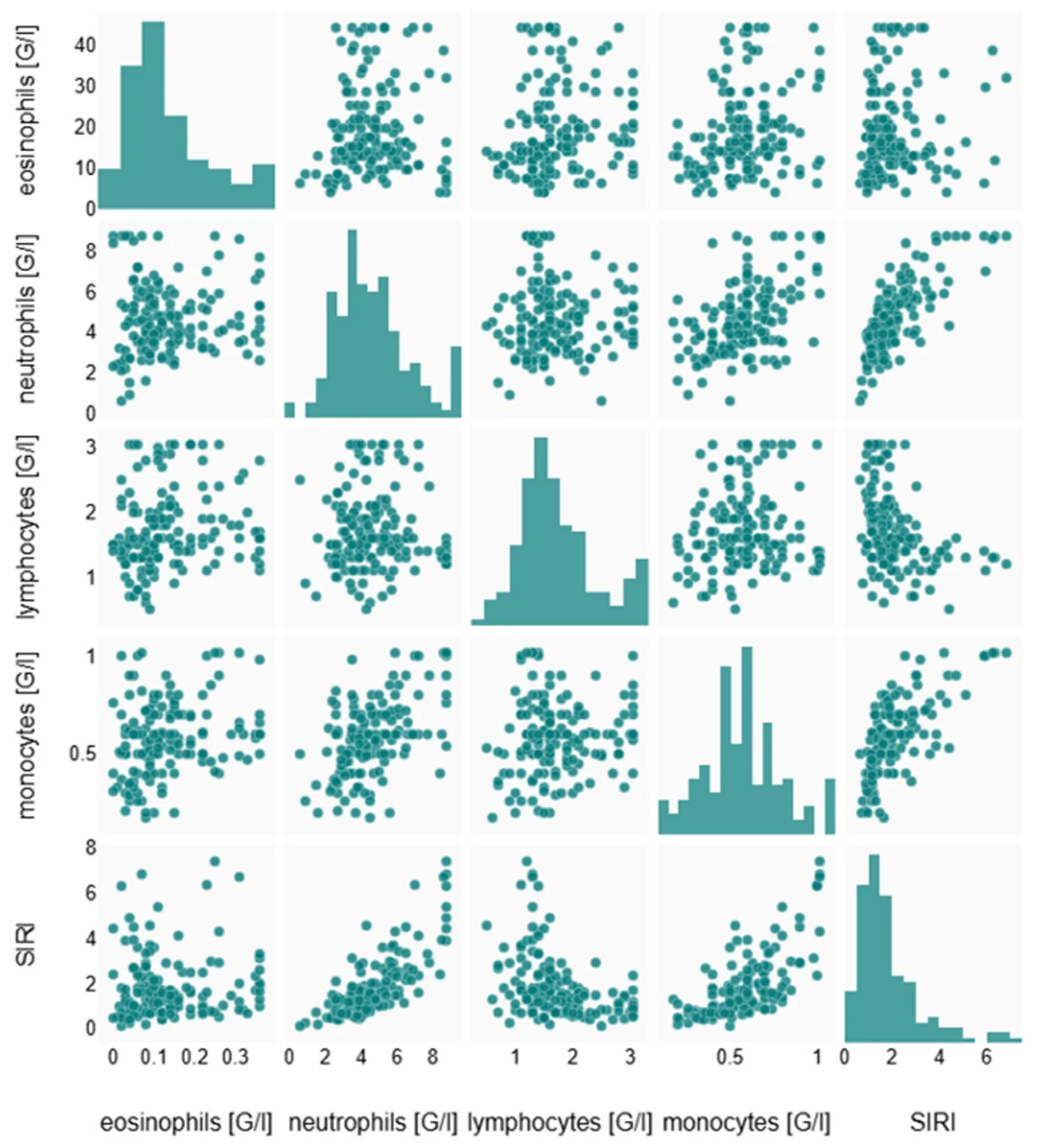
Illustration of how raw blood morphology parameters affect the SIRI: low SIRI values occur with both low and high LYMPH counts (r = 0.25, *p* = 0.002); the SIRI is linearly associated with MONO (r = 0.66, *p* = 0.000); the association between the SIRI and NEU (r = 0.75, *p* = 0.000) may reflect the correlation between MONO and NEU; and no associations were observed between the SIRI and EOS (r = 0.07, *p* = 0.384).

Immunophenotyping can be understood as identifying specific features of a patient’s immune profile. For example, investigators have examined PD-1 and PD-L1 expression in relation to checkpoint inhibitors [14], investigated immune-evasion mechanisms involving altered CD58 expression [15], and proposed broader frameworks for describing tumor immunity, such as “inflamed,” “immune-excluded,” and “immune-desert” phenotypes [16]. This approach is central to precision medicine because it aims to identify targets for treatment. However, these complex assessments require considerable expertise and resources. The immune phenotype of an individual patient remains incompletely understood, despite the low cost of basic tests such as a complete blood count and assessment of circulating immune cells. Blood count-derived ratios, including NLR, MLR, and SIRI, have been widely investigated; our results for these ratios are presented in Table 1, Table 2, Table 3, Table 4, Table 5, Table 6 and Table 7 and, particularly, in Figure 7. Figure 7 illustrates the relationships between the SIRI and selected raw blood count parameters. Mathematically, the SIRI has several limitations: LYMPH is the denominator, whereas the numerator is the product of NEU and MONO; NEU and MONO are correlated; and NEU values are generally much higher than LYMPH or MONO values. Consequently, the SIRI is positively correlated with NEU and MONO, potentially repeating information contained in these counts, and negatively correlated with LYMPH. However, low SIRI values may occur with both low and high LYMPH counts, and the SIRI is not correlated with EOS. The NLR and the MLR have analogous limitations. Together with the fact that these ratios are not routinely used to guide clinical decisions, this supports exploring alternative approaches to interpreting immunological biomarkers. SAMBA offers a way to interpret blood morphology in a more nuanced manner while making the rationale for classification transparent and retaining potentially informative raw data. Retaining raw values is important for statistical interpretation and clinical practice: neutropenia is relevant when assessing chemotherapy complications, eosinophilia is important in T2-associated diseases, and lymphocytosis is relevant in hematology. Raw blood cell counts may also provide information about independent or coordinated biological processes. In this cohort, for example, neutrophil and monocyte counts were correlated, as were eosinophil and basophil counts; these relationships may reflect shared pathways of proliferation or activation. The use of ratios alone does not justify disregarding the underlying values. Given the retrospective design, the relatively small and heterogeneous cohort, and the lack of independent validation, our findings should be considered preliminary. The proposed phenotypic labels are exploratory and hypothesis-generating. They are based partly on laboratory reference ranges and partly on hypotheses generated by the observed outcomes, which require further verification. We propose the following preliminary interpretations: the exhausted phenotype may reflect marked tumor-associated inflammation or concomitant infection and could be associated with immune-cell dysfunction; the lymphopenic phenotype may indicate reduced antitumor or antiviral immune capacity, potentially related to tumor-induced cytokines or impaired lymphopoiesis; the eosinopenic (T2-low) phenotype may reflect a relatively balanced inflammatory profile with limited T2-associated immune activity; and the eosinophilic (T2-high) phenotype may occur without major abnormalities in other white blood cell counts. Changes in immune status, including malnutrition-associated lymphopenia, may also contribute to differences in prognosis. A transparent, readily interpretable algorithm could help standardize and accelerate preliminary research in immuno-oncology. Studies that assess both the tumor microenvironment and comorbidities may help explain these observations and establish whether the proposed classification has clinical utility. Those obvious relations are usually not highlighted in publications regarding blood count-derived markers [17,18,19,20,21,22,23,24,25,26,27,28,29,30,31].

### Study Limitations

This retrospective study has several limitations. First, patient recruitment was not optimal because we excluded patients who had received preoperative chemotherapy to ensure that blood morphology results were not affected by treatment. Second, the cohort was relatively small and heterogeneous in disease stage. We could not obtain data on postoperative treatment, which may have been administered at specialized oncology centers or omitted. Although the primary aim was to propose a transparent approach to classification rather than to establish a definitive prognostic model, independent validation remains necessary. CART provides a simplified classification; survival-tree methods might fit the data better but are less accessible to analysts with limited statistical programming experience and were not available in the STATISTICA package used. We therefore used CART while explicitly acknowledging its limitations. The proposed classification is exploratory and does not constitute independent validation. Overfitting may have occurred because the SAMBA phenotypes were derived and evaluated in the same cohort. Finally, the novelty of the SAMBA approach and the lack of directly comparable studies limit the context for interpreting our findings.

## 5. Conclusions

First, blood count parameters in patients with PDAC may be associated with survival, as absolute white blood cell counts may partly reflect inflammatory processes in tissues, including the tumor and its microenvironment. Second, more detailed characterization of the immune status of patients with PDAC is warranted. Indices such as the NLR may be useful for general risk stratification; however, because they combine multiple parameters, they cannot distinguish, for example, neutrophilia with a normal lymphocyte count from a normal neutrophil count with lymphopenia. Thus, these indices may omit information that is relevant to immunological interpretation.

Although several aspects of our findings remain unclear, further immunological studies in oncology cohorts may improve patient classification. Much remains to be learned, particularly through large-scale studies, and immunophenotyping may ultimately contribute to personalized cancer care.

## Figures and Tables

**Table 1:** General characteristics of the study group.

	n	%
**Stage**		
**IA**	2	1.30%
**IB**	15	9.80%
**IIA**	19	12.42%
**IIB**	25	16.34%
**III**	7	4.58%
**IV**	85	55.56%
**Grade**		
**1**	28	18.30%
**2**	104	67.97%
**3**	21	13.73%
**Localization**		
**Head**	111	72.55%
**Body/tail**	42	27.45%
**Resection**		
**R0**	52	61.18%
**R1**	16	38.83%
**Gender**		
**Female**	86	56.21%
**Male**	67	43.79%
**6-month survival**		
**Yes**	86	56.21%
**No**	67	43.79%
**12-month survival**		
**Yes**	56	36.60%
**No**	97	63.40%
	**n**	**Median (min.–max.); IQR**
**Age [years]**	153	65.0 (46.0–85.0); 59.0–70.0
**NEU [G/L]**	153	4.30 (0.60–8.76); 4.5–5.6
**LYMPH [G/L]**	153	1.60 (0.50–3.05); 1.30–2.00
**MONO [G/L]**	153	0.60 (0.17–1.02); 0.49–0.70
**EOS [G/L]**	153	0.11 (0.00–0.36); 0.06–0.18
**BASO [G/L]**	153	0.03 (0.01–0.07); 0.02–0.04
**NLR**	153	2.53 (0.24–8.60); 1.82–3.75
**MLR**	153	0.33 (0.11–1.06); 0.24–0.46
**CRP [mg/L]**	83	4.50 (0.60–37.66); 2.23–16.40
**SIRI**	153	1.47 (0.12–7.40); 0.93–2.32
**Ca 19-9 [U/mL]**	153	148.40 (0.60–1000.00); 21.38–500.00
**Ca 125 [U/mL]**	153	19.20 (4.00–66.85); 12.60–34.30
**CEA [U/mL]**	153	3.14 (0.30–12.65); 1.90–6.20
**Ca 15-3 [U/mL]**	153	20.60 (2.20–40.15); 14.90–25.00

**Table 2:** Characteristics of study subgroups according to eosinophil (EOS) and basophil (BASO) levels, according to the laboratory reference ranges.

	EOS ≤0.05 G/L	EOS > 0.05 G/L	*p*	BASO < 0.02 G/L	BASO 0.02–0.06 G/L	BASO > 0.06 G/L	*p*
**Stage**			0.570				0.637
**I**	4 (12.5%)	13 (10.7%)		5 (9.1%)	11 (12.8%)	1 (8.3%)	
**II**	10 (31.3%)	34 (28.1%)		19 (34.6%)	24 (27.9%)	1 (8.3%)	
**III**	0 (0.0%)	7 (5.8%)		2 (3.6%)	4 (4.7%)	1 (8.3%)	
**IV**	18 (56.3%)	67 (55.4%)		29 (52.7%)	47 (54.7%)	9 (75.0%)	
**Grade**			0.186				0.651
**1**	3 (9.4%)	25 (20.7%)		11 (20.0%)	14 (16.3%)	3 (25.0%)	
**2**	26 (81.3%)	78 (64.5%)		37 (67.3%)	61 (70.9%)	6 (50.0%)	
**3**	3 (9.4%)	18 (14.9%)		7 (12.7%)	11 (12.8%)	3 (25.0%)	
**Localization**			0.427				0.078
**Head**	25 (78.1%)	86 (71.1%)		34 (61.8%)	67 (77.9%)	10 (83.8%)	
**Body/tail**	7 (21.9%)	35 (29.9%)		21 (38.2%)	19 (22.1%)	2 (16.7%)	
**Resection**			0.228				0.770
**R0**	9 (64.3%)	43 (79.6%)		21 (80.8%)	29 (74.4%)	2 (66.7%)	
**R1**	5 (35.7%)	11 (20.4%)		5 (19.2%)	10 (25.6%)	1 (33.3%)	
**Gender**			0.693				0.380
**Female**	17 (53.1%)	69 (57.0%)		29 (52.7%)	52 (60.5%)	5 (41.7%)	
**Male**	15 (46.9%)	52 (43.0%)		26 (47.3%)	43 (39.5%)	7 (58.3%)	
**6-Month survival**			0.110				0.611
**Yes**	14 (43.8%)	72 (59.5%)		28 (50.9%)	51 (59.3%)	7 (58.3%)	
**No**	18 (56.3%)	49 (40.5%)		27 (49.1%)	35 (40.7%)	5 (41.7%)	
**12-Month survival**			0.126				0.860
**Yes**	8 (25.0%)	48 (39.7%)		21 (38.2%)	30 (34.9%)	5 (41.7%)	
**No**	24 (75.0%)	73 (60.3%)		34 (61.8%)	56 (65.1%)	7 (58.3%)	
**Age [years]**	63.0	66.0	0.508	64.0	66.0	67.0	0.920
**CRP [mg/L]**	4.5	4.5	0.147	4.5	4.6	3.0	0.963
**SIRI**	1.1	1.6	0.089	1.3	1.5	1.8	0.165
**Ca 19-9 [U/mL]**	257.3	138.4	0.142	195.5	120.3	269.6	0.410
**Ca 125 [U/mL]**	15.9	19.4	**0.044**	18.8	19.2	30.1	0.281
**CEA [U/mL]**	2.9	3.2	0.346	2.9	3.1	3.6	0.576
**Ca 15-3 [U/mL]**	20.6	20.6	0.470	20.6	20.6	22.7	0.159
**Overall survival**	4.5	8.0	0.146	7.0	8.0	7.0	0.885

**Table 3:** Characteristics of study subgroups according to monocyte (MONO) and lymphocyte (LYMPH) levels, according to the laboratory reference ranges.

	MONO < 0.31 G/L	MONO 0.31–0.81 G/L	MONO > 0.81	*p*	LYMPH <1.1 G/L	LYMPH 1.1–2.5 G/L	LYMPH > 2.5 G/L	*p*
**Stage**				0.913				0.646
**I**	1 (8.3%)	14 (11.1%)	2 (13.3%)		1 (4.6%)	13 (11.8%)	3 (14.3%)	
**II**	5 (4.7%)	36 (28.6%)	3 (20.0%)		6 (11.8%)	34 (30.9%)	4 (19.1%)	
**III**	0 (0.0%)	6 (4.8%)	1 (6.7%)		0 (0.0%)	6 (5.5%)	1 (4.8%)	
**IV**	6 (50.0%)	70 (55.6%)	9 (60.0%)		15 (68.2%)	57 (51.8%)	13 (61.9%)	
**Grade**				0.379				0.057
**1**	3 (25.0%)	23 (12.3%)	2 (13.3%)		1 (4.6%)	22 (20.0%)	5 (23.8%)	
**2**	9 (75.0%)	89 (68.3%)	9 (60.0%)		19 (86.4%)	75 (68.2%)	10 (47.6%)	
**3**	0 (0.0%)	17 (13.5%)	4 (26.7%)		2 (9.1%)	6 (11.8%)	6 (28.6%)	
**Localization**				0.676				0.806
**Head**	10 (83.8%)	90 (71.4%)	11 (73.3%)		16 (72.7%)	81 (73.6%)	14 (66.7%)	
**Body/tail**	2 (16.7%)	36 (28.6%)	4 (26.7%)		6 (27.7%)	29 (26.4%)	7 (33.3%)	
**Resection**				0.209				0.054
**R0**	3 (50.0%)	45 (80.4%)	4 (66.7%)		4 (57.1%)	44 (83.0%)	4 (50.0%)	
**R1**	3 (50.0%)	11 (19.6%)	2 (33.3%)		3 (42.9%)	9 (17.0%)	4 (50.0%)	
**Gender**				0.046				**0.038**
**Female**	8 (66.7%)	74 (58.7%)	4 (26.7%)		10 (45.5%)	59 (53.6%)	17 (80.9%)	
**Male**	4 (33.3%)	52 (41.3%)	11 (73.3%)		12 (54.6%)	51 (46.4%)	4 (19.1%)	
**6-Month survival**				**0.041**				**0.044**
**Yes**	6 (50.0%)	76 (60.3%)	4 (26.7%)		7 (31.8%)	66 (60.0%)	14 (61.9%)	
**No**	6 (50.0%)	50 (39.7%)	11 (73.3%)		15 (68.2%)	44 (40.0%)	8 (38.1%)	
**12-Month survival**				0.130				**0.011**
**Yes**	4 (33.3%)	50 (39.7%)	2 (13.3%)		2 (9.1%)	47 (42.7%)	7 (33.3%)	
**No**	8 (66.7%)	76 (60.2%)	13 (86.7%)		20 (90.9%)	63 (57.3%)	14 (66.7%)	
**Age [years]**	62.5	65.0	67.0	0.642	66.0	65.5	65.0	0.892
**CRP [mg/L]**	12.4	3.8	37.7	**0.002**	3.8	12.5	3.0	**0.041**
**SIRI**	0.7	1.5	4.3	**<0.001**	1.5	2.0	1.0	**<0.001**
**Ca19-9 [U/mL]**	153.1	148.4	84.8	0.826	152.6	277.7	50.1	0.344
**Ca125 [U/mL]**	12.7	19.2	19.4	0.090	19.2	19.2	16.6	0.389
**CEA [U/mL]**	2.9	3.1	3.1	0.506	3.1	3.1	3.5	0.627
**Ca15-3 [U/mL]**	20.1	20.6	21.4	0.945	20.6	20.8	20.0	0.676
**Overall survival**	6.5	8.0	1.0	**0.004**	4.5	8.5	9.0	**0.025**

**Table 4:** Characteristics of study subgroups according to neutrophil (NEU) levels and literature-based cutoffs for the neutrophil-to-lymphocyte ratio (NLR) and monocyte-to-lymphocyte ratio (MLR).

	NEU ≤ 6.1 G/L	NEU > 6.1 G/L	*p*	NLR ≤ 5	NLR > 5	*p*	MLR ≤ 0.3	MLR > 0.3	*p*
**Stage**			0.065			0.786			0.612
**I**	16 (12.4%)	1 (4.2%)		16 (12.1%)	1 (4.8%)		6 (15.0%)	11 (9.7%)	
**II**	40 (31.0%)	4 (16.7%)		38 (28.8%)	6 (28.6%)		13 (32.5%)	31 (27.4%)	
**III**	4 (3.1%)	3 (12.5%)		6 (4.6%)	1 (4.8%)		1 (2.5%)	6 (5.3%)	
**IV**	69 (53.5%)	16 (66.7%)		72 (54.6%)	13 (61.9%)		20 (50.0%)	65 (57.5%)	
**Grade**			0.464			0.834			0.443
**1**	23 (17.8%)	5 (20.8%)		25 (18.2%)	4 (19.1%)		10 (25.0%)	18 (15.9%)	
**2**	90 (69.8%)	14 (58.3%)		89 (67.4%)	15 (71.4%)		25 (62.5%)	79 (69.9%)	
**3**	16 (12.2%)	5 (28.8%)		19 (14.4%)	2 (9.5%)		5 (12.5%)	16 (14.2%)	
**Localization**			0.429						
**Head**	92 (71.3%)	19 (79.2%)							
**Body/tail**	37 (28.7%)	5 (20.8%)							
**Resection**			0.434			0.321			0.417
**R0**	45 (75.0%)	7 (87.5%)		47 (78.3%)	5 (62.5%)		14 (70.0%)	38 (79.2%)	
**R1**	15 (25.0%)	1 (12.5%)		13 (21.7%)	3 (37.5%)		6 (30.0%)	10 (20.8%)	
**Gender**			0.265			0.023			**0.005**
**Female**	75 (58.1%)	11 (45.8%)		79 (59.9%)	7 (33.3%)		30 (75.0%)	56 (49.6%)	
**Male**	54 (41.9%)	13 (54.2%)		53 (40.1%)	14 (66.7%)		10 (25.0%)	57 (50.4%)	
**6-Month survival**			**0.014**			**0.006**			**0.041**
**Yes**	78 (60.5%)	8 (33.3%)		80 (60.6%)	6 (28.6%)		28 (70.0%)	58 (51.3%)	
**No**	51 (39.5%)	16 (66.7%)		52 (39.4%)	15 (71.4%)		12 (30.0%)	55 (48.7%)	
**12-Month survival**			**0.027**			**0.072**			0.199
**Yes**	52 (40.3%)	4 (16.7%)		52 (92.9%)	4 (19.1%)		18 (45.0%)	38 (33.6%)	
**No**	77 (59.7%)	20 (83.3%)		80 (60.6%)	17 (80.9%)		22 (55.0%)	75 (66.4%)	
**Age [years]**	66.0	64.0	0.228	66.0	47.0	0.553	62.5	66.0	0.193
**CRP [mg/L]**	4.1	10.6	**0.020**	1.4	0.6	0.015	3.6	4.8	0.575
**SII**	603.0	1277.1	**<0.001**	796.7	50.4	**<0.001**	389.3	745.1	**<0.001**
**SIRI**	1.32	3.6	**<0.001**	1.1	0.1	**<0.001**	0.7	1.7	**<0.001**
**Ca19-9 [U/mL]**	148.4	96.0	0.596	0.6	0.6	0.096	51.8	205.6	**0.010**
**Ca125 [U/mL]**	18.8	19.6	0.083	4.0	4.0	0.100	13.3	20.8	**<0.001**
**CEA [U/mL]**	3.1	3.4	0.924	1.4	0.4	0.363	3.1	3.1	0.588
**Ca15-3 [U/mL]**	20.6	19.6	0.419	5.4	2.2	0.718	19.2	20.7	0.056
**Overall survival**	9.0	3.5	0.109	3.0	8.5	**0.077**	7.0	11.0	0.220

**Table 5:** Correlations between continuous blood morphology parameters. Correlations with r > 0.4 were considered moderate and flagged (bolded) for further assessment.

	NEU [G/L]	LYMPH [G/L]	MONO [G/L]	EOS [G/L]	BASO [G/L]
**NEU [G/L]**	-	R = 0.03; *p* = 0.680	**R = 0.46; *p* = 0.000**	R = 0.08; *p* = 0.271	R = 0.24; *p* = 0.002
**LYMPH [G/L]**	R = 0.03; *p* = 0.680	-	R = 0.09; *p* = 0.255	R = 0.24; *p* = 0.002	R = 0.20; *p* = 0.011
**MONO [G/L]**	**R = 0.46; *p* = 0.000**	R = 0.09; *p* = 0.255	-	R = 0.24; *p* = 003	R = 0.34; *p* = 0.000
**EOS [G/L]**	R = 0.08; *p* = 0.271	R = 0.24; *p* = 0.002	R = 0.24; *p* = 003	-	**R = 0.48; *p* = 0.000**
**BASO [G/L]**	R = 0.24; *p* = 0.002	R = 0.20; *p* = 0.011	R = 0.34; *p* = 0.000	**R = 0.48; *p* = 0.000**	-

**Table 6:** Comparison of clinical characteristics across the proposed blood morphology phenotype subgroups.

	Exhausted	Lymphopenic	Eosinopenic T2-Low	Optimal T2-High	*p*
**Overall survival [months]**	1.5	4.5	8.0	10.5	<0.001
**Stage**					0.786
**I**	2; 16.7%	1; 4.6%	3; 12.0%	11; 11.7%	
**II**	3; 25.0%	6; 27.3%	7; 28.0%	28; 29.8%	
**III**	1; 8.3%	0; 0%	0; 0%	6; 6.4%	
**IV**	6; 50.0%	15; 68.2%	15; 60.0%	49; 52.1%	
**Grade**					0.194
**1**	2; 16.7%	1; 4.6%	3; 12.0%	22; 23.4%	
**2**	7; 58.3%	19; 86.4%	20; 80.0%	58; 61.7%	
**3**	3; 25.0%	2; 9.1%	2; 8.0%	14; 14.9%	
**Localization**					0.948
**Head**	8; 66.67%	16; 72.73%	19; 76.00%	68; 72.34%	
**Body/tail**	4; 33.33%	6; 27.27%	6; 24.00%	26; 27.66%	
**Resection**					0.417
**R0**	4; 67.7%	4; 57.1%	7; 70.0%	37; 82.2%	
**R1**	33.3%	3; 42.9%	3; 30.0%	8; 17.8%	
**Gender**					0.192
**Female**	4; 33.3%	10; 45.5%	14; 56.0%	58; 61.7%	
**Male**	8; 66.7%	12; 54.6%	11; 44.0%	36; 38.3%	
**6-Month survival**					0.003
**Yes**	3; 25.0%	7; 31.8%	14; 56.0%	62; 66.0%	
**No**	9; 75.0%	15; 68.2%	11; 44.0%	32; 34.1%	
**12-Month survival**					0.003
**Yes**	2; 16.7%	2; 9.1%	8; 32.0%	44; 46.8%	
**No**	10; 83.3%	20; 90.9%	17; 68.0%	50; 53.2%	
**Median (IQR)**					
**Age [years]**	67.0 (62.5–69.0)	65.5 (60.0–71.0)	66.0 (59.0–73.0)	65.0 (59.0–70.0)	0.925
**CRP [mg/L]**	37.7 (22.1–37.7)	12.5 (4.5–21.9)	2.6 (1.5–5.9)	3.2 (2.0–10.6)	<0.001
**NEU [G/L]**	7.50 (6.00–8.75)	4.05 (3.40–5.20)	3.80 (2.70–5.30)	4.30 (3.50–5.30)	<0.001
**LYMPH [G/L]**	1.50 (1.30–2.10)	1.00 (0.80–1.10)	1.50 (1.40–2.10)	1.87 (1.50–2.10)	<0.001
**MONO [G/L]**	0.95 (0.90–1.01)	0.50 (0.34–0.70)	0.50 (0.37–0.60)	0.60 (0.50–0.68)	<0.001
**EOS [G/L]**	0.14 (0.06–0.26)	0.08 (0.06–0.10)	0.03 (0.02–0.4)	0.14 (0.10–0.22)	<0.001
**BASO [G/L]**	0.04 (0.04–0.07)	0.02 (0.02–0.03)	0.02 (0.01–0.03)	0.03 (0.02–0.05)	<0.001
**NLR**	4.71 (3.27–6.43)	4.32 (3.18–5.60)	2.00 (1.69–2.87)	2.33 (1.79–3.00)	<0.001
**MLR**	0.63 (0.44–0.75)	0.51 (0.6–0.70)	0.28 (0.21–0.41)	0.32 (0.24–0.41)	<0.001
**SIRI**	4.39 (2.91–6.48)	2.00 (1.31–3.12)	1.07 (0.60–1.73)	1.41 (0.87–1.80)	<0.001
**Ca19-9 [U/mL]**	70.4 (20.1–330.7)	277.7 (34.5–500.0)	243.6 (26.6–1000.0)	139.3 (19.7–500.0)	0.549
**Ca125 [U/mL]**	19.2 (12.4–35.6)	19.2 (15.2–42.1)	16.1 (10.1–24.7)	19.3 (12.6–34.8)	0.550
**CEA [U/mL]**	3.4 (1.8–5.2)	3.1 (1.9–10.2)	2.7 (2.0–6.4)	3.2 (2.0–5.9)	0.913
**Ca15-3 [U/mL]**	20.0 (10.2–30.9)	20.8 (17.3–24.8)	20.0 (14.5–22.7)	20.6 (14.9–26.4)	0.689

**Table 7:** Univariate Cox proportional hazards regression models. Reference categories (HR = 1): stage IV, grade 3, body/tail tumor location, R1 resection, male sex, lymphopenic phenotype, NEU ≤ 6.1 G/L, LYMPH 1.1–2.5 G/L, MONO 0.31–0.8 G/L, BASO ≤ 0.02 G/L, and EOS > 0.05 G/L.

Parameter (Effect Level)	HR (95%CI)	*p*
**Stage**		
**I–III**	0.55 (0.39—0.78)	0.001
**Grade**		
**1**	0.68 (0.37–1.22)	0.192
**2**	0.76 (0.47–1.23)	0.263
**Localization**		
**Head**	1.23 (0.84–1.79)	0.287
**Resection**		
**R0**	0.57 (0.32–1.02)	0.058
**Gender**		
**Female**	1.05 (0.75–1.46)	0.79
**Phenotypes: Exhausted**	1.03 (0.50–2.14)	0.933
**Eosinopenic**	0.50 (0.27–0.90)	0.022
**Optimal (T2-high)**	0.38 (0.23–0.62)	<0.001
**NEU**		
**>6.1**	0.52 (0.97–2.39)	0.067
**LYMPH**		
**≤1.1**	2.31 (1.43–3.72)	0.001
**>2.5**	0.95 (0.57–1.57)	0.830
**MONO**		
**<0.31**	1.33 (0.74–2.42)	0.343
**>0.8**	2.45 (1.40–4.29)	0.002
**EOS**		
**≤0.05**	0.72 (0.48–1.09)	0.118
**BASO**		
**0.2–0.6**	1.84 (0.85–3.98)	0.124
**>0.6**	0.75 (0.41–1.36)	0.343
**NLR**		
**>5**	0.60 (0.37–0.97)	0.036
**MLR**		
**<0.3**	0.79 (0.54–1.16)	0.227
**Age [years]**	1.01 (0.99–1.03)	0.349
**CRP [mg/L]**	1.01 (1.00–1.03)	0.089
**SIRI**	1.0 (1.0–1.0)	0.100
**Ca19-9 [U/mL]**	1.0 (1.0–1.0)	0.100
**Ca125 [U/mL]**	1.01 (1.00–1.02)	0.018
**CEA [U/mL]**	1.03 (0.98–1.08)	0.197
**Ca15-3 [U/mL]**	0.99 (0.97–1.01)	0.524

**Table 8:** Multivariate Cox proportional hazards regression model (*p* = 0.003). Correlated variables were excluded. Reference categories (HR = 1): stage IV, grade 3, body/tail tumor location, male sex, and lymphopenic phenotype.

Parameter (Effect Level)	HR (95%CI)	*p*
**Stage**		
**I–III**	0.74 (0.51–1.09)	0.129
**Grade**	0.67 (0.36–1.23) 0.77 (0.45–1.33)	
**1**		0.196
**2**		0.351
**Localization**	1.24 (0.81–1.88)	
**Head**		0.32
**Gender**	1.11 (0.78–1.69)	
**Female**		0.656
**Phenotype**	1.15 (0.51–2.59) 0.48 (0.25–0.89) 0.42 (0.25–0.70)	0.737 0.02 0.001
**Exhausted**		
**Eosinopenic**		
**Optimal (T2-high)**		
**Age [years]**	1.01 (0.98–1.03)	0.565
**Ca19-9**	1.00 (1.00–1.00)	0.012
**Ca125**	1.01 (1.00–1.02)	0.204
**CEA**	1.01 (0.96–1.01)	0.742
**Ca15-3**	0.99 (0.96–1.01)	0.211

**Table 9:** Best-fitting multivariate Cox proportional hazards regression model (*p* = 0.000; stepwise selection). Reference categories (HR = 1): stage IV, grade 3, and lymphopenic phenotype.

Parameter (Effect Level)	HR (95%CI)	*p*
**Stage I–III**	0.63 (0.45–0.90)	0.010
**Phenotype**		
**Exhausted**	1.14 (0.56–2.34)	0.718
**Eosinopenic**	0.48 (0.27–0.88)	0.017
**Optimal (T2-high)**	0.41 (0.25–0.88)	0.017
**Ca19-9**	1.01 (1.00–1.01)	0.004

## Data Availability

Data available upon a reasonable request.

## Supplementary Materials

The following supporting information can be downloaded at: https://www.mdpi.com/article/10.3390/cancers18213477/s1, Table S1, Post-hoc results (only for statistically significant comparisons; Kruskal-Wallis test with Bonferroni correction): MONO and LYMPH parameters.; Table S2, Post-hoc results (only for statistically significant comparisons; Kruskal-Wallis test with Bonferroni correction): blood morphology phenotypes subgroups.
