# Supplementary material for: Simple Algorithm for Blood Morphology Analysis (SAMBA): An Artificial Intelligence-Assisted Approach to Identify Peripheral Blood Phenotypes in a Cohort of Patients with Pancreatic Ductal Adenocarcinoma (PDAC)
Source: Cancers (Basel). 2026 Oct 28;18(21):3477. doi: 10.3390/cancers18213477 (PMC13762038; doi:10.3390/cancers18213477)
Supplement: Supplementary file 1 [file cancers-18-03477-s001.zip › cancers-4587500-supplementary.pdf]

cancers

MDPI

Supplementary Materials: Simple Algorithm for Blood Morphology Analysis (SAMBA): An Artificial Intelligence-Assisted Approach to Identify Peripheral Blood Phenotypes in a Cohort of Patients with Pancreatic Ductal Adenocarcinoma (PDAC)

Aleksander Wardęszkiewicz, Konrad Kosztowny, Oliwia Grząsiak-Kraj, Piotr Hogendorf, Adam Durczyński, Anna Kumor-Kisielewska, Michał Panek, Janusz Strzelczyk and Alicja Majos

Table S1. Post-hoc results (only for statistically significant comparisons; Kruskal-Wallis test with Bonferroni correction): MONO and LYMPH parameters.

	**MONO: CRP**		
	MONO <0,31 G/l	MONO 0,31-0,81 G/l	MONO >0,81
**MONO <0,31 G/l**		0,608	0,450
**MONO 0,31-0,81 G/l**	0,608		0,003
**MONO >0,81**	0,450	0,003	
	**MONO: SIRI**		
	MONO <0,31 G/l	MONO 0,31-0,81 G/l	MONO >0,81
**MONO <0,31 G/l**		0,001	0,000
**MONO 0,31-0,81 G/l**	0,001		0,000
**MONO >0,81**	0,000	0,000	
	**MONO: Overall survival**		
	MONO <0,31 G/l	MONO 0,31-0,81 G/l	MONO >0,81
**MONO <0,31 G/l**		1,000	0,283
**MONO 0,31-0,81 G/l**	1,000		0,003
**MONO >0,81**	0,283	0,003	
	**LYMPH: CRP**		
	LYMPH <1,1 g/L	LYMPH 1,1-2,5 G/l	LYMPH >2,5 G/l
**LYMPH <1,1 g/L**		0,035	0,822
**LYMPH 1,1-2,5 G/l**	0,035		1,000
**LYMPH >2,5 G/l**	0,822	1,000	
	**LYMPH: SIRI**		
	LYMPH <1,1 g/L	LYMPH 1,1-2,5 G/l	LYMPH >2,5 G/l
**LYMPH <1,1 g/L**		0,258	0,000
**LYMPH 1,1-2,5 G/l**	0,258		0,003
**LYMPH >2,5 G/l**	0,000	0,003	
	**LYMPH: Overall survival**		
	LYMPH <1,1 g/L	LYMPH 1,1-2,5 G/l	LYMPH >2,5 G/l
**LYMPH <1,1 g/L**		0,021	0,149
**LYMPH 1,1-2,5 G/l**	0,021		1,000
**LYMPH >2,5 G/l**	0,149	1,000	

Table S2. Post-hoc results (only for statistically significant comparisons; Kruskal-Wallis test with Bonferroni correction): blood morphology phenotypes subgroups.

		**CRP**		
	Exhausted	Lymphopenic	Eosinopenic T2-low	Optimal T2-high
**Exhausted**		1,000	0,061	0,019
**Lymphopenic**	1,000		0,010	0,003
**Eosinopenic T2-low**	0,061	0,010		1,000
**Optimal T2-high**	0,019	0,003	1,000	
		**NEU**		
	Exhausted	Lymphopenic	Eosinopenic T2-low	Optimal T2-high
**Exhausted**		0,000	1,000	1,000
**Lymphopenic**	0,000		0,000	0,000
**Eosinopenic T2-low**	1,000	0,000		1,000
**Optimal T2-high**	1,000	0,000	1,000	
		**LYMPH**		
	Exhausted	Lymphopenic	Eosinopenic T2-low	Optimal T2-high
**Exhausted**		0,000	0,000	0,000
**Lymphopenic**	0,000		1,000	1,000
**Eosinopenic T2-low**	0,000	1,000		1,000
**Optimal T2-high**	0,000	1,000	1,000	
		**MONO**		
	Exhausted	Lymphopenic	Eosinopenic T2-low	Optimal T2-high
**Exhausted**		0,000	0,568	1,000
**Lymphopenic**	0,000		0,000	0,000
**Eosinopenic T2-low**	0,568	0,000		0,417
**Optimal T2-high**	1,000	0,000	0,417	
		**EOS**		
	Exhausted	Lymphopenic	Eosinopenic T2-low	Optimal T2-high
**Exhausted**		0,921	0,003	0,003
**Lymphopenic**	0,921		1,000	0,000
**Eosinopenic T2-low**	0,003	1,000		0,000
**Optimal T2-high**	0,003	0,000	0,000	
		**BASO**		
	Exhausted	Lymphopenic	Eosinopenic T2-low	Optimal T2-high
**Exhausted**		0,021	0,086	1,000
**Lymphopenic**	0,021		0,767	0,002
**Eosinopenic T2-low**	0,086	0,767		0,003
**Optimal T2-high**	1,000	0,002	0,003	

		**NLR**		
	Exhausted	Lymphopenic	Eosinopenic T2-low	Optimal T2-high
**Exhausted**		1,000	0,000	0,000
**Lymphopenic**	1,000		0,001	0,004
**Eosinopenic T2-low**	0,000	0,001		1,000
**Optimal T2-high**	0,000	0,004	1,000	
		**MLR**		
	Exhausted	Lymphopenic	Eosinopenic T2-low	Optimal T2-high
**Exhausted**		1,000	0,000	0,000
**Lymphopenic**	1,000		0,000	0,000
**Eosinopenic T2-low**	0,000	0,000		1,000
**Optimal T2-high**	0,000	0,000	1,000	
		**SIRI**		
	Exhausted	Lymphopenic	Eosinopenic T2-low	Optimal T2-high
**Exhausted**		0,071	0,054	0,017
**Lymphopenic**	0,071		0,000	0,000
**Eosinopenic T2-low**	0,054	0,000		1,000
**Optimal T2-high**	0,017	0,000	1,000	
		**Overall survival**		
	Exhausted	Lymphopenic	Eosinopenic T2-low	Optimal T2-high
**Exhausted**		1,000	0,007	0,530
**Lymphopenic**	1,000		0,003	0,148
**Eosinopenic T2-low**	0,007	0,003		1,000
**Optimal T2-high**	0,530	0,148	1,000	
